# Whole-Genome Sequence of *Lactobacillus sakei* LT-13 Isolated from Moto Starter of Sake

**DOI:** 10.1128/genomeA.00651-17

**Published:** 2017-08-03

**Authors:** Shiro Kato, Tadao Oikawa

**Affiliations:** a High Technology Research Core, Kansai University, Suita, Osaka, Japan; b Faculty of Chemistry, Materials, and Bioengineering, Kansai University, Suita, Osaka, Japan

## Abstract

*Lactobacillus sakei* strain LT-13 is a lactic acid bacterium isolated from moto starter of Japanese sake. This genome analysis revealed that the genome is composed of a circular chromosome and one plasmid, which contain 1,938 and 8 putative protein-coding genes, respectively.

## GENOME ANNOUNCEMENT

Fermented foods are an important constituent of human life in various areas of the world, and many lactic acid bacteria are used for the production of various fermented foods (1, 2). *Lactobacillus* is the largest genus of lactic acid bacteria, and studies about *Lactobacillus* have focused on various aspects, such as its genomics and metabolomics (3). *L. sakei* is a lactic acid bacterial species of the *Lactobacillus* genus, and it plays an important role in the traditional Japanese sake fermentation process (4). *L. sakei* can release some metabolites, such as amines, amino acids, organic acids, and sugars, into growth medium (5). Some of these metabolites affect the taste of the fermented product (6). Here, we report the complete genome sequence of *L. sakei* LT-13 isolated from moto starter of sake.

The genomic DNA of strain LT-13 was grown in de Man-Rogosa-Sharpe (MRS) broth and extracted using a DNeasy blood and tissue kit (Qiagen), according to the manufacturer’s protocol. DNA libraries for shotgun sequencing (400-bp read) and paired-end sequencing (8-kb span) were constructed using a GS Titanium rapid library preparation kit (Roche) and GS Titanium libraries of paired-end adaptors (Roche). The whole-genome sequencing was performed with a GS Junior 454 sequencer (Roche), and the shotgun and paired-end reads were assembled using the GS De Novo Assembler version 2.9. Gap filling was carried out by the Sanger sequencing method. Prediction and annotation of the coding sequences were performed using the Microbial Genome Annotation Pipeline (MiGAP) (7).

The GS reads were assembled into 2 scaffolds: one consists of 30 large contigs for the chromosome, and another is for the plasmid, and the genome coverage was approximately 71-fold. The total length of the strain LT-13 chromosome was 1,936,922 bp, with a G+C content of 41.17%. The sequence of a 6,214-bp plasmid, with a G+C content of 35.97%, was also determined. The numbers of putative protein-coding sequences predicted and annotated by MiGAP were 1,938 and 8 for the chromosome and plasmid, respectively. The chromosome contained 64 and 15 coding sequences for tRNAs and rRNAs, respectively. The genomic properties of strain LT-13, including chromosome size and the number of genes, were similar to those of the *L. sakei* strain 23K isolated from fresh sausage (8). We hope that the presented genome data will be helpful for studies on the genomics and metabolomics of *L. sakei* and on food fermentation with *L. sakei*.

### Accession number(s).

The complete genome sequence has been deposited in DDBJ under the GenBank accession numbers AP017929 (chromosome) and AP017930 (plasmid).
